# Humans use minimum cost movements in a whole-body task

**DOI:** 10.1038/s41598-021-99423-5

**Published:** 2021-10-11

**Authors:** Lijia Liu, Dana Ballard

**Affiliations:** Department of Computer Science, The University of Texas at Austin, Austin, TX USA

**Keywords:** Computational science, Computer modelling, Control theory

## Abstract

Humans have elegant bodies that allow gymnastics, piano playing, and tool use, but understanding how they do this in detail is difficult because their musculoskeletal systems are extraordinarily complicated. Nonetheless, common movements like walking and reaching can be stereotypical, and a very large number of studies have shown their energetic cost to be a major factor. In contrast, one might think that general movements are very individuated and intractable, but our previous study has shown that in an arbitrary set of whole-body movements used to trace large-scale closed curves, near-identical posture sequences were chosen across different subjects, both in the average trajectories of the body’s limbs and in the variance within trajectories. The commonalities in that result motivate explanations for its generality. One explanation could be that humans also choose trajectories that are economical in cost. To test this hypothesis, we situate the tracing data within a forty eight degree of freedom human dynamic model that allows the computation of movement cost. Using the model to compare movement cost data from nominal tracings against various perturbed tracings shows that the latter are more energetically expensive, inferring that the original traces were chosen on the basis of minimum cost.

## Introduction

Movements are arguably the most important reason for the evolution of the brain; living things that do not move do not have brains^1^. The human brain and its movement system, in particular, have a huge co-dependency in development that starts in the womb and continues throughout childhood^2^. Thus elucidating the structure of movements cannot but held give us information of the brain’s structure and vice versa.

The properties of the human vision system can give us a hint of what could be at stake in this partnership. The premier finding in the brain’s cortical visual memory is that not all possible images are coded, only the most common and useful. This observation raises the possibility for the movement system. Are only the most common and useful movements coded in the motor cortex? If this possibility turned to be true, it could lead to a revolution in how we think about the cortex’s coding of movement. Ergo this question provides a sine qua non for understanding movement costs.

However, in getting started, it is important to acknowledge there is one way movements are not like vision as several different optimization principles must be considered. One is that the musculoskeletal system must deal with newton’s equations in describing the movement’s dynamics. These bring their features as candidates for principles, e.g., Jerk as a model for torques^3^. Another issue is that human cognition can influence how important are a human’s momentary goals in selecting their movements. For example, rewarding reaches can modulate the trajectories^4^. However, despite these alternatives, the omnipresence of the earth’s gravitational field motivates our nervous system to choose trajectories that are economical in energetic cost^5,6^.

Another point to make is that although our focus is on the cost of movements, we did not analyze the movements enough to see just how the model came up with mix of joint torques. This focus, particularly how the body can change its mix during complex movements, is studied in modular approaches e.g.^7^ We eschewed modular analysis on our study, but believe that our model is not only compatible with modular models but that they have precepts that could lead to helpful movement codings.

Studying the human movement system is daunting owing to its complexity. Its skeletomuscular system has about 350 joints and over 650 muscles.This reason accounts for the observation that most previous studies of movement have focused on common movements. Humans’ self-selected trajectories or posture sequences are economical in energetic cost has been shown in simple single-behavior motions such as walking^8–10^, running, and reaching^11,12^, with attention especially given to walking because it is rhythmic and easy to study. Furthermore, for example,the system can adapt preferred gaits to minimize energetic cost in response to varying loads^13–15^.

Experiments shave also shown that humans’ walking speed^16^, step frequency/length^17–23^, step width^24,25^ all correlate with the minimum metabolic cost. Most importantly, energetic cost exhibits a classic U-shaped dependence on cost wherein the minimums define a preferred parameter, here the step frequency while walking at a constant speed^10,21^.

However, whether the principle is true for large-scale whole-body movements, that call for unfamiliar posture sequences, was still an open question. An answer was suggested by, our complex whole-body virtual tracing experiment^26^ that aimed to learn principles behind *large-scale arbitrary* movements, particularly regarding variations between different subjects.

In that study, a full-body virtual-reality curve tracing task elicited a series of human movement sequences^26^. Participants traced three dimensional space curves and their posture sequences were continuously recorded using a motion-capture system. Special aggregation methods made possible by the joint limits of the biological system were developed. Theses methods allowed data analysis that extracted similarities of posture sequences in the face of kinematic variations. Given Bernstein’.s classic comments on the impossibility of reproducing movements exactly^27^, our result was exciting and unsuspected. Both the movement’s posture sequences and kinematic variances showed striking commonalities across different subjects.

The obvious inference from the observed similarities of movements across different subjects is that there must be some general principle for humans’ motion commonalities. This regularity of movements across different subjects implies energetic cost should be similar and provides motivation for tour experiments that test this hypothesis.

For the energetic cost computation test, we took advantage of a special forty-eight degrees of freedom dynamic computational model capable of simulating, analyzing, and synthesizing humanoid movements^28^. The model consists of twenty-one body components connected by twenty joints and incorporates several novel features. One innovation is that the joint connections are not treated as perfectly rigid constraints but rather as very stiff springs that hold body parts together like tendons and muscles. The model allows computing instantaneous power from the product of net joint torque and joint angular velocity. The work performed at each joint was determined by numerically integrating the instantaneous powers over the entire tracing task. In this way, the energetic cost of human motions can be computed given motion capture data.

### Test overview

Given the observation that multiple subjects generating large-scale movements^26^ used identical postures, we could use our dynamics model to compute the cost of each of the posture sequences. The goal was to test if the subjects’ posture sequences were somehow minimal in cost. The test we chose to illustrate this concept was perturbation analysis. This method changes the original tracing protocol into a similar protocol by adding small changes. Next, each subject’s model was used to trace the curve using the perturbed protocol. The costs of different subjects’ curve traces were computed and compared to the costs of tracing the original curve. Two different kinds of perturbations were used. In one, the tracing trajectories were slightly perturbed by shifting positions of a particular body part of the dynamic model a small amount for the duration of the trace. In the other, the original tracing path was displaced in certain small increments prior to the trace. The result of both of these kinds of perturbations was that their energetic costs means were higher than those of the original curve tracings. The rationale is that in the original trace the subject had the freedom to choose a posture sequence with minimum cost, while in the perturbed tracing protocol, they were forced to use a different posture sequence as the perturbations added external constraints so that the experimental data was revealed to be more expensive. Therefore, the energetic costs exhibited a classical U-shape with respect to the different posture sequences, with the minimum of the U-shape curve consistent with the cost of the original posture traces. These results strongly suggest that movement is selected on the basis of minimum cost.

## Background

In the past two decades the study of motion cost has seen a revolution with the development of direct integration techniques that are sufficiently accurate to be used for individual subjects. To motivate their value, we briefly review some of their antecedent techniques.

In the past, a common way to address the minimum energetic cost principle was to conduct experiments comparing walking and running with many other strange and unpractised gaits ^29,30^. Three commonly used methods are still used to study energy optimization.

The most straightforward and frequently used method is to measure the metabolic cost, e.g., subjects breath through a mouthpiece to measure oxygen consumption rates (VO2). For example, subjects were required to walk under different circumstances, and the results showed that the metabolic cost was minimum while subjects walked at the condition which was “comfortable” for them^13–19^. The advantage of this method is that movements can be related directly to energetic cost, but the measuring apparatus is typically very constraining.

A common way to measure muscle activation and stiffness (co-activation) is to use Electromyography (EMG). Huang et al.^31^ showed that that subjects’ metabolic cost is reduced during the learning process of arm reaching tasks, and their muscle activities and co-activation would parallel changes in metabolic power. However, EMG measures just correlates that need additional modeling to turn them into an energetic cost.

A third method, dynamic modeling, is to build a closed-form analytical mechanics-based model and determine if the predicted minimum mechanical cost correlates with people’s kinematic preferences. For example^20–22,24^ use an inverted pendulum model to predict the optimal step length and compare it with the subjects’ natural step length while walking.

All these methods face obstacles for calculating the energetic cost of whole-body tracing movements collected from the VR experiment^26^. These methods are time-consuming, and the required configuration restricts the variety of experiments. For example, the VO2 process does not work for our virtual-reality tracing tasks as subjects need to wear the VR helmet on their head, leaving little space for a mouthpiece. Besides, the EMG method measures muscle co-contraction, which is correlated with energetic cost, rather than calculating the cost. Another way is to build a humanoid dynamic model. The method is a good way to imitate human movements, and it is widely used in biomedical engineering due to its compliance with real-world physical rules. However, it has several critical limitations: (1) it is too difficult to model and control a complex system, such as a whole human body. (2) it is challenging to represent “kinematic loops,” such as postures that need both feet are on the ground. (3) for large systems, the equations of motion in nested, rotating reference frames become very complex, making them more challenging to approximate well. Due to the complexity and disadvantages of the dynamic modeling method for large complex systems, most of the studies took advantage of two-dimensional models to study human part-body motions in the sagittal plane.

Still other methods build a two dimensional dynamic bipedal robot by modeling the whole body with a skeleton of rigid segments connected with joints. However, those methods over-simplify human bodies so that they can only study simple single-behavior human movements. The simplest bipedal robot uses three links to represent the torso and two legs in the sagittal plane ^32,33^. Five-link biped robots extend the model using two links to represent each leg^34–37^, while seven-link biped robots further extend it by adding feet to it^38,39^. Furthermore, those methods have many assumptions while studying human locomotion. For example, most researchers assume that instantaneous exchange of the biped support legs occurs when the swing leg contacts with the ground. In this way, the biped locomotion with single foot support can be considered as a successive open loop of kinematic chain from the support point to the free ends, as robot manipulators. Recently, three dimensional modeling of biped robots^40,41^ has been developed. However, they are still not sophisticated enough compared with a real human body.

In the face of these complex challenges, a major alternate modeling route is to incorporate a complete muscoskeletal models. One of the most complete is that of OpenSim^42–44^, which allows modeling detail to the level of attached muscles. However this level can be difficult to handle in building a generate model so we chose to focus on a simpler dynamic model at the level of joint torques and inertias and model more abstract versions of the human system that still use multiple degrees of freedom but summarize muscle effects through joint torques.

The computation of the dynamics of such multi-jointed systems recently has also experienced significant advances. The foremost of these, use a kinematic plan to integrate the dynamic equations directly. Several different dynamic libraries exist, such as MuJoCo^45^, Bullet^46^, Havok^47^, Open Dynamic Engine(ODE)^48^, and PhysX^49^ (A simplified version of OpenSim can also be used). An evaluation of several systems by^50^ found them roughly comparable in capability.

### An individual subject model

Our Human Dynamic Model (HDM) system^28^ is built on top of the physics engine ODE, the most commonly used dynamic library in the robotic area. The 48 degree of freedom model is also based on a direct integration method. It has a singular focus on individual human movement modeling and uses a unique approach to integrating the dynamic equations. A direct dynamics integration method to extracts torques from human subjects in real-time^51–53^ using a unifying spring constraint formalism.

These toques have two components. The major component is the one determined by the open-loop integration of Newton’s equations. These must be supplemented by a closed-loop set of “residual torques” to achieve accurate balance. This organization models the similar dichotomy in the human system.

At each frame, instantaneous power was computed from the product of the net joint torque and joint angular velocity. The work performed at each joint was determined by numerically integrating the instantaneous powers over the entire tracing task. In this way, given motion capture data, we can compute the mechanical cost without building a humanoid biped robot with motion equations. Note that it is common to use mechanical measures of work to indicate the metabolic energy consumption^54^. The “energetic cost” mentioned in the following sections means the mechanical cost. The HDM dramatically simplifies the process of calculating movement cost and extends the potential kinds of movements that can be studied, but it has two limitations: (1) the motion capture data must be given; (2) the model size needs to be tuned each time fitting a subject. An extensive validation of this dynamic model appears in^28^.

### Experiment data preview

While doing the virtual tracing experiment, subjects freely chose their starting posture and were given no instructions on how to perform themselves. Therefore, participants were tracing curves at their preferred posture sequences. In other words, they traced curves under the conditions which were “comfortable” for them. According to the previous experiments^13–19^, we can expect that the energetic costs of movements with those trajectories should be a minimum or at least locally minimum. To establish our conclusion, the cost of original virtual tracing movements and perturbed movements were computed and compared using the human dynamic model. As expected, the energetic cost always exhibits a U-shape while tracing using different postures sequences, with the minimum of the U-shape curve consistent with the original posture traces. In this way, we are able to demonstrate the energetic cost of original trajectories is a local minimum. The method section describes the experimental protocol in more detail.

## Results

**Figure 1:**
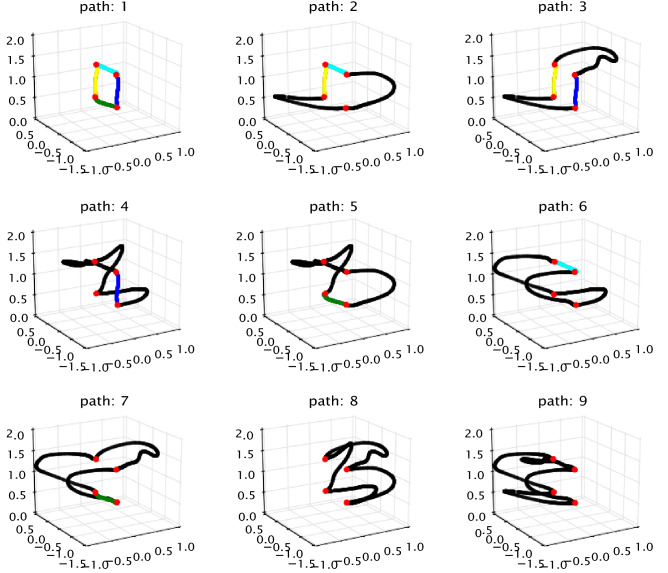
The nine 3-dimensional paths in the virtual environment that were used in the experiment. They are ordered by their complexity. For reference, colors denote common segments and points. For the subjects, the paths were all rendered in black, The scale is in meters.

**Figure 2:**
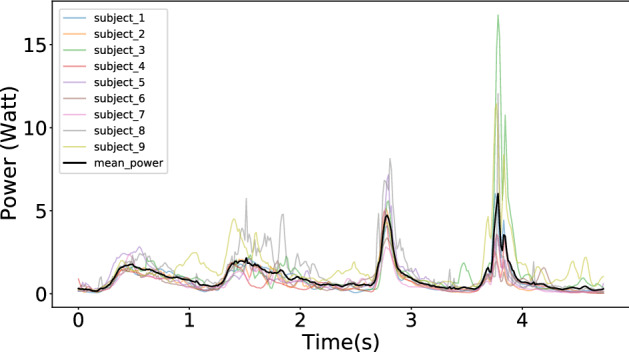
The power of tracing path1 at each frame. Nine subjects traced path1 five times. The plot shows the average joints’ power at each frame for each of the subjects in a color. The black line indicates the mean. The common curve similarities indicate that different subjects had similar power patterns while tracing the same curve, which shows that each subject has difficulty in tracing at the same point in the trace. Path 1 is the most straightforward, but the observation of correlated effort represents patterns in tracing other curves.

**Figure 3:**
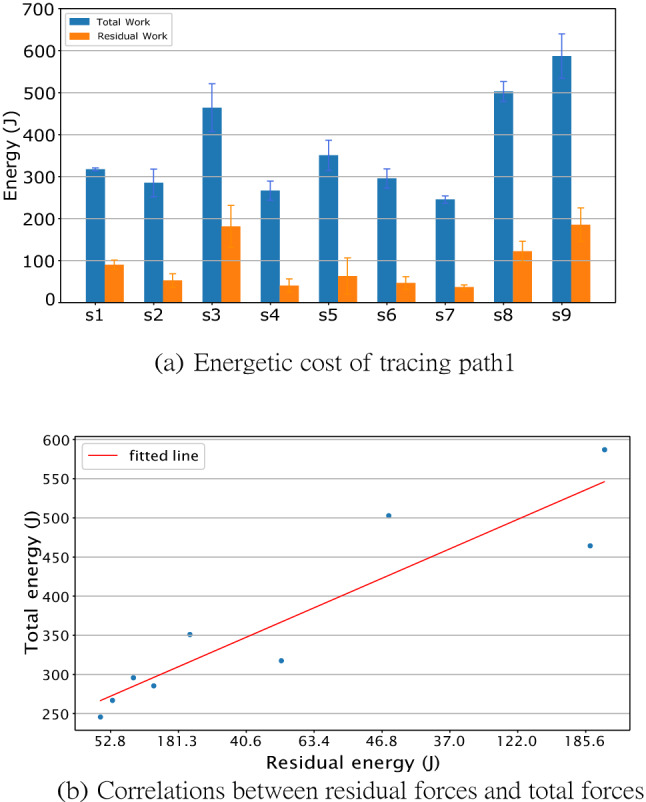
Energetic costs of tracing path 1. (**a**) Each subject traced path 1. with five repeats. The horizontal labels indicate the related subjects, e.g., “S1” represents the subject1. The total cost is shown in blue, and the portion of that cost due to residual forces are shown in orange. A low cost in residual torque usually signifies that the dynamic model is a good match for that subject’s kinematic data. (**b**) The residual is highly correlated with the overall cost (R=0.922), suggesting that the differences in both values reflect differences in the body models.

**Figure 4:**
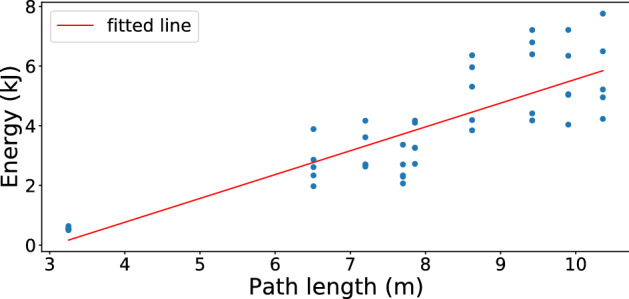
The energy cost of tracing nine paths. These results portray the possibility that the costs vary across the best-fit five subjects. The statistics show that each path traced has a unique cost based on its length that distinguishes it from the rest. The correlation between the length and cost of tracing shows a R value of 0.85.

**Figure 5:**
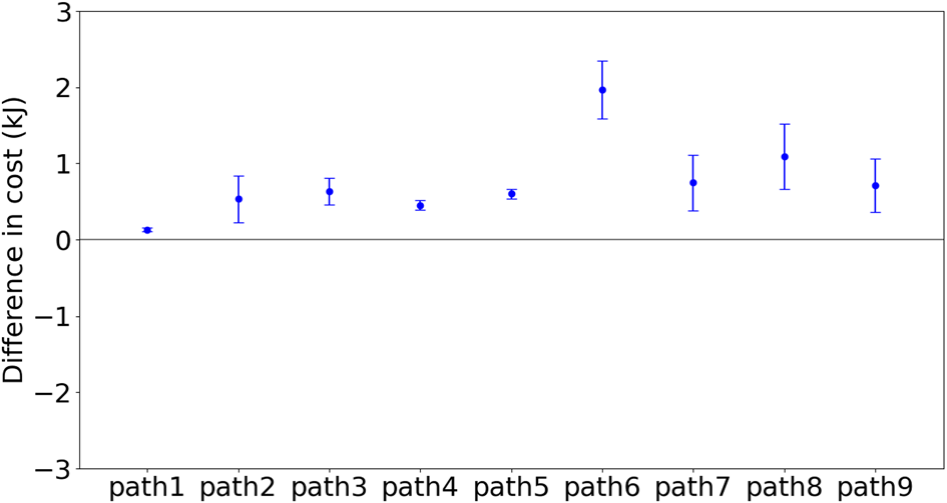
The difference in the energetic cost for the model perturbation. The figure shows the difference in the energetic cost of tracing each of the nine paths with perturbations in the right-elbow marker. The elbow was moved up 5cm. The results show that the original path is always the least expensive for all the paths and the averages across subject tracers. Moreover, the differences between the energetic costs of original trajectories and perturbed trajectories are highly significant, with a p-value less than 0.001.

**Figure 6:**
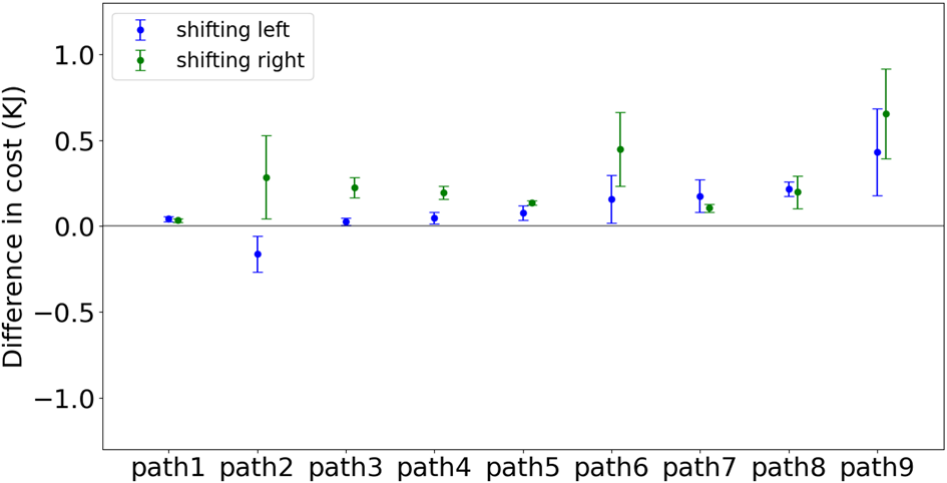
The difference in the energetic cost for the path perturbation. Each of the nine paths has two perturbations of 5 cm: left in blue, right in green. This main result shows that the original path is always the least expensive for both averages across subject traces. The difference in the energetic cost for the path perturbation is not very clear but still reliable, with a p-value less than 0.01.

**Figure 7:**
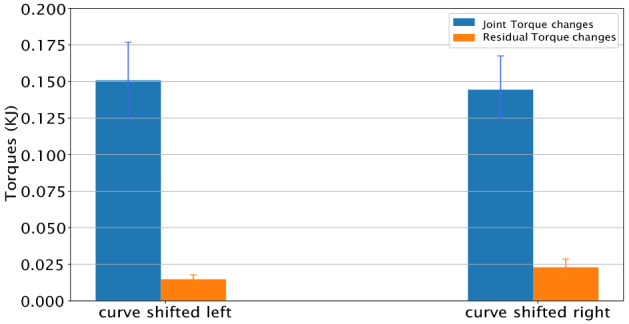
Torque changes. The average of the means of the cost changes for path 1 with five repeats across five participants.

Using the kinematic curve tracing data from^26^, we fitted the dynamic model to each of the eighteen subjects and then had the models trace the nine curves that are shown in Fig. 1. The energy cost of tracing paths showed several marked regularities:

1. The joints’ power allocation while tracing path1 across different subjects showed that although the total costs of the movements varied between subjects, the temporal power use was qualitatively very similar.(See Fig. 2);
2. The computation of average energy cost when nine subjects traced path1 five times showed that the magnitude of the required residual forces for each subject was relatively small. (See Fig. 3);
3. The costs of tracing each path by each subject were very similar and approximately monotonic with the length of paths. (See Fig. 4);
4. Although there are variations in the cost across the repeated traces, tracing with the perturbed model parameters was significantly higher than the original. (See Figs. 5 and  6);
5. The increased energy cost while using perturbed model parameters depended much more on the joints’ cost than on the residual component. (See Fig. 7);

### The mean of power across different participants

As an initial analysis, we established the variations in the energetic costs for tracing path1 exhibited by different subjects. Fig. 2 illustrates the mean and the standard deviation of powers across subjects at each frame. The result reveals that subjects put similar effort at the same points along the path. Thus although the total cost of the movements may vary between subjects, the power patterns are qualitatively very similar. The VR experiment^26^ showed that participants used similar postures sequences while tracing the same curves from a kinematic perspective. It is expected that the instantaneous power of joints at each frame should be similar as well due to the skeleton constraints of the human body. The similarity of power patterns across different subjects reinforces this conclusion from a dynamics perspective.

Although there are qualitative similarities in the difficult points on the curve, the total costs of the traces differ across different subjects. This result is expected due to the variety of subjects’ skeletons and weights.

### Residual forces

In the human system, the computed torques cannot be depended on to be exact but have to be supplemented by residual torques. This residual is most prominently due to the vestibular and proprioception systems. The human dynamic model has to deal with the same issue. The recovered torques are close to exact but leave a small residual. To deal with it, we implemented a improvised system of torques (See “Methods” section). As shown in Fig. 3, the highest cost of the tracing movement is the component owing to the joint torques. The total energy of tracing a path1, including the residual components, is shown in blue, and the residual component is shown separately in orange. When reporting the energetic costs of the traces, we always use the total cost in blue.

Furthermore, the ratio of the residual and total cost can be used as a criterion to decide whether the HDM body segment dimensions are a good fit for the raw motion capture data. As shown in Fig. 3, some subjects are fitting better than others, e.g. the residual of subject 3 and subject 9 are relatively high. Therefore, the five best-fitting subjects (s2, s4, s5, s6, s7) were chosen for all the following experiments. Subsequently, we noticed a correlation (r = 0.922) in the data. All subjects have a very similar ratio between the residual and total cost implying that we were looking at effects of a body parameter such as weight. Based on this observation, we felt comfortable using only the subjects with the smallest residuals, as the rest are extremely unlikely to make a difference.

### Energy cost of tracing nine paths

Although there are similar energetic costs per subject in tracing an identical path, this arrangement does not carry over to the comparison between paths, which has larger per subject differences. Instead, we hypothesized that the average costs should scale as the length of the path, as shown in Fig. 4, which shows the energetic cost of tracing the nine different paths. The paths differ in tracing cost, but the costs of tracing each path by each subject are very similar and approximately monotonic with the lengths of the paths.

Given these regularities, the next step was to evaluate the significance of perturbations in the tracing protocol. The hypothesis is that if the tracing postures are chosen to be of minimum energy, changing the configuration away from the actual tracing situation should incur a cost, which was what happened.

### Model perturbation

The first perturbation test changed model marker trajectories. Specifically, the right-elbow marker was shifted up or down by a small delta on every traced curve,which produced a new constraint that the model needed to satisfy while tracing paths. Once the delta was chosen, it was used for each frame in the trace. To implement it, the dynamic model had to trace paths using identical posture sequences except for lifting its right elbow. Although kinematics of the body parts except the right elbow remained for the unperturbed trace – only the kinematics of the right elbow changed, the joints’ constraints bias the dynamic model adapt to follow the new perturbed trace.

For each trace, the right-elbow marker was raised by 5 cm. The rest of the system adapted the way dictated by the dynamic constraints. Fig. 5 shows the difference in cost of constrained motions and original motions. It is seen that although there are variations in the cost across the repeated traces, the cost of using the perturbed model is higher than the original. Note that outside of the changes, the rest of the model solves the inverse dynamic model with the unperturbed parameters, and thus the model has substantial degrees of freedom at its proposal. The t-test showed the difference of the probability distributions between the original cost and the perturbed cost is reliable, with a p-value less than 0.001. Furthermore, the increase of tracing complex paths is larger than that of tracing simple paths.

### Path perturbation

The second perturbation test made adjustments in the traced path, called path perturbation. Some effects of displacement can be intuited. For example, if a subject has to reach over their head during the trace, it can be expected that lowering the traced path would result in cost savings. For this reason, we chose path perturbations in the horizontal plane. Two such perturbations were used: a 5-centimeter leftward displacement and a 5-centimeter rightward displacement. Left and right are referenced to the coordinate system used for the four points used for all nine curves (See Fig. 1).

In this way, new constraints were produced as the dynamic model was required to trace the perturbed paths while the starting tracing positions were not changed. In contrast to the model perturbation, the model’s trace paths were shifted while the posture sequences remain the same. Again, the dynamic model took advantage of internal joint constraints to adjust original posture sequences to trace the perturbed paths.

Figure 6 shows the difference in average energetic costs for tracing displaced paths and original paths across subjects. The blue dots indicate the difference between tracing left-shifted paths and tracing the original path, while the green dots represent the other case. For most cases, the original paths are seen to be consistent with the lowest cost. Path 2 with 5cm leftward displacement costs less than the original path 2. The reason is that subjects preferred to stand near the left corner, where is the starting tracing position. However, the left part of path 2 is much easier than its right part (See Fig. 1). Therefore, when shifting path 2 to the left, subjects became closer to the right part, which led to easier tracing. In contrast, subjects had to move their bodies more to trace well when shifting path 2 to the right.

Here again, The overall result is an endorsement of our hypothesis that the unconstrained traces are minimal. If that is true, we should see that all the perturbed traces are more costly, which happened. Although some overlap, the original paths are more economical for almost all curves than the displacements. The significant test showed the effects of shitting paths is not very clear but still reliable, with a p-value less than 0.01. The observation that the averages of all the perturbed costs are larger than the average cost of their original progenitors strongly suggests that energy cost is the factor in the choice of tracing postures.

### Residual forces

Given the dynamics dichotomy, a natural question that arises concerns the magnitude of the extra torques in the perturbation cases. Are the extra costs carried by the dynamic model or the residual? It can be answered by interrogating the simulation, and it turns out that the dynamics model’s contribution is dominating. This is shown in Fig 7.

Note that if the constraints on the dynamics were highly stiff, then the model would have no course other than tracing an exact copy of the unperturbed trajectory and let the residual torques contribute the needed difference. However, the markers on the body for these experiments were limited to 15–18 of key body segments, leaving the extra degrees of freedom to be determined by the dynamics. Moreover, the torque computation, to model the reality of muscles^55^, used spring constraints at each joint degree of freedom. Finally, the right finger was required to contact the displaced paths, and the remaining features of the movement are the same, leaving the dynamics to fill in the rest.

## Discussion

Given that the cost of the movements is a significant fraction of a human’s caloric budget^56^, one might expect that humans would exhibit common low-cost postures but arbitrary whole-body movements have been less studied, so the expectations are open. Thus it was a surprise to measure arbitrary movements in a large-scale tracing task and find markedly common posture sequences used by all tested subjects^26^. Several alternatives have been offered to explain movements e.g.^57–59^ but compared to these and other alternatives, which tend to be specialized, our minimal energetic cost hypothesis enjoys its simplicity and the adjunct rationale of the ubiquity of the gravitational field, especially when there were no complex constraints in the movements and no constraints on the time to perform the traces.

Our simulation extends the kinematic finding to show that tests of human dynamics provide evidence that movements are chosen on the basis of energetic economic costs. As the cost of tracing scales monotonically with the length of a traced path as expected, the possibility is raised that segments of the trajectories are also minimal. We did not test this possibility but it is important as if was true, it would open the possibility of creating arbitrary curves by composing appropriate segments.

As already mentioned the complex human movement system relies on residual forces, as would be expected from the human’s vestibular system and others so they are a potential confound of the result. However as shown in Fig. 7, these were relatively small, with similar magnitudes in the different perturbations.

The main quantitative results are that subjects’ traces of each of nine space paths all have minimal costs with respect to local perturbations. The manipulation introducing perturbations in the kinematic variables were the subjects traced the path but their model with small displacements in a kinematic marker was straightforward. However, in the other experiment, the local displacements of the paths were constrained to be horizontal. Verticals were not used as they can be equivocal. The displacements can interact with the different body heights, e.g., a short subject has to reach an uncomfortable height. However, outside of this caveat, all the data can be interpreted as the tracing posture sequences selected based on energetic cost.

The hypothesis that humans use minimum cost movement trajectories is shown by the use of a human dynamic model that leverages a significant innovation in dynamics computation that allows the recovery of torques from kinematic data. A limit of the current method is that we perturbed motions manually, so it is possible that we found only a local minimum in the space of possible movements. However, as tracing a path usually takes more than 1000 frames, and 50 markers represent a posture at each frame, the perturbation space searched was large.

One very significant possibility raised by the local minimum result is that the trajectories are pre-computed and stored^60–62^. This possibility is reinforced with by progress in the sparse coding of temporal sequences^63,64^ that strongly suggest that trajectories are remembered to obviate the difficulties of computing them online. And if movements are to be stored, the less expensive ones are likely to be preferred^65^.

How this could be done and even if it could be done are open questions. A future possibility is to study an algorithm with the capability of sequential potential long movements automatically, such as reinforcement learning, incorporating the constraints between possible posture segments.

## Methods

### Virtual tracing experiment

**Figure 8:**
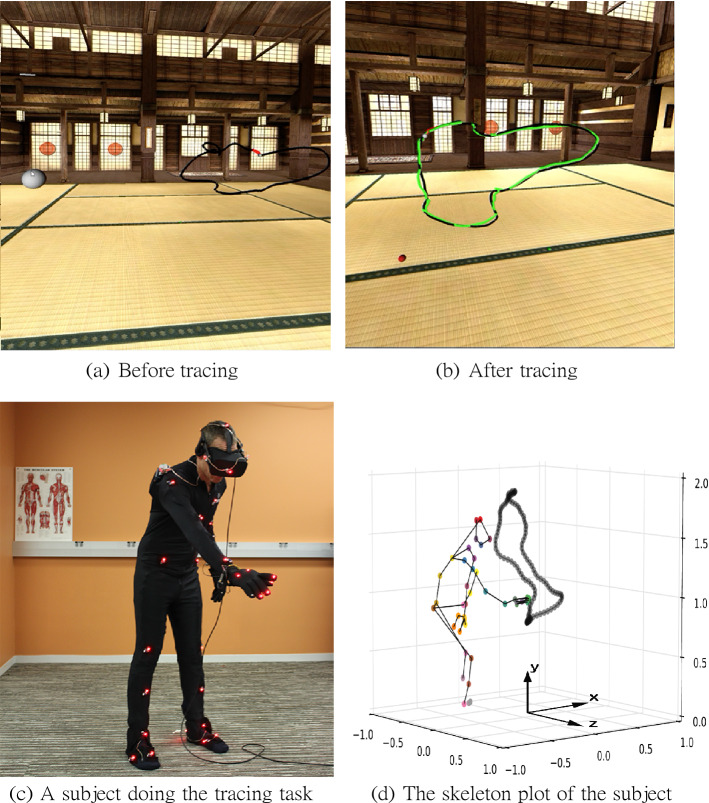
The virtual environment setup^26^. (**a**) Shows a full view of a path, denoted by a black path, and the starting position, denoted by a large white sphere. The small white sphere on the path at the end of a red segment is the tracing target sphere. (**b**) Depicts the scene when a trial is finished. The green path is the actual tracing trajectory generated by a subject. (**c**) Illustrates a subject in the act of tracing a path in the laboratory’s motion capture 2 × 2 × 2 meter volume and (**d**) shows the lab coordinate system. The scale on the graph is in meters. The the subject’s skeleton and the traced path in the 3D space are plotted. The color dots correspond to a subset of the fifty active-pulse LED markers on the suit and the virtual-reality helmet.

The original kinematic data capture were collected from a virtual whole-body tracing experiment that was to elicit natural movements under common goals^26^. Subjects wore a virtual-reality helmet, Oculus Rift^66^, to see a virtual three dimensional interior room with a dojo backdrop via stereo video. They were required to trace a series of paths positioned at fixed locations in the virtual environment. The experiment protocol forces subjects to trace each curve from a fixed starting position and use a fixed velocity, thus trials for all subjects tracing one curve have the same time frames. Except for these constraints, subjects freely chose their initial postures and were given no instructions on how to comport themselves during the tracing process. The extent of curves is large enough so that subjects need to move their legs to complete a trace. The movements of their bodies and variables relevant to the tasks were simultaneously recorded using the PhaseSpace motion capture system^67^. The WorldViz Vizard software package^68^ both controlled the virtual tracing protocol and the recording of the motion capture data. Fig. 8 shows the virtual environment setup.

#### Data post-processing

For some frames the motion capture system is unable to determine the 3-dimensional location of some markers, thus raw motion capture data usually contains some segments of signal loss (dropouts). Dropouts are relatively infrequent in practice but can occur over significant temporal intervals, which makes linear interpolation a poor choice for reconstructing the raw motion capture data. In this experiment, trajectory-based singular value threshold was implemented to reconstruct missing marker data with a minimal impact on its statistical structure. The data for each subject was interpolated using a separate matrix completion model.

In addition to the data interpolation process, if a participant did not trace the path successfully we would consider this tracing invalid and the data unusable. Because if a recording of a tracing trial failed, e.g., too many markers were off during a tracing, it will lead to extremely large joint torques, which is unrealistic.

#### Statement

The Institutional Review Board (IRB) at the University of Texas at Austin approved the experiments; This experiment were performed in accordance with relevant named guidelines and regulations; And informed consent was obtained from all participants and/or their legal guardians.

### Computational modeling

#### Human dynamic model details

In developing a human model there are many choices. Even in modeling at this level there are several choices given alternative models for integrating the dynamics equations^50^. Our choice is a model^52,53^ built on top of ODE^48^ that incorporates additional features added for human systems:

- Anthropomorphic joint models use soft constraints for their joints that allow some local movement.Such constraints would allow us to simulate co-contraction at the joint level, but in our calculations this constraint is not modelled for two reasons. One is that we assume that if our subjects are choosing inexpensive movements, they will try and minimize the co-contraction. The other more important reason is that in our perturbation analyses co-contraction measures should cancel.
- Significant innovations have been added in order to handle the closed-loop kinematic chains of bipedal movements and the contact constraints they introduce, which have proven difficult to model.
- Toques calculationshave two components. The major component is the one determined by the open-loop integration of Newton’s equations. These must be supplemented by a closed-loop set of “residual torques” to achieve accurate balance. This organization models the similar dichotomy in the human system.

These features give the human dynamic model (HDM)^28^ a singular focus on human movement modeling and comprise a unique approach to integrating the dynamic equations. The direct dynamics integration method to extracts torques from human subjects in real-time^51–53^ using a unifying spring constraint formalism.

#### Model topology

**Figure 9:**
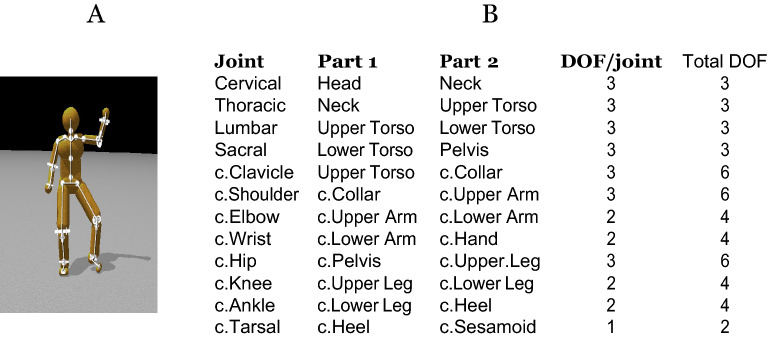
The 48 internal DOF Model^28^. (**A**) Four ball-and-socket joints connect five body-segments along the spine from the head to the waist. Ball-and-socket joints are also used at the collar-bone, shoulder, and hip. (**B**) A summary of the joints used in the model. c. = chiral: there are two of each of these joints (left and right). Universal joints are used at the elbows, wrists, knees, and ankles. Hinge joints connect the toes to the heels. All joints limit the range of motion to angles plausible for human movement. Our model assumes that joint DOFs summarize the effects of component muscles.

To compute the energy cost of subjects tracing paths, we used our human dynamic model^28^. By replaying the virtual tracing experiment’s kinematic data, we can compute can the joints’ properties, e.g. torques and angles, at frame rates. The human dynamic model is built on top of the ODE physics engine^69^. It consists of a collection of rigid bodies connected by joint. Each joint connects two rigid bodies with anchor points (center of rotation) defined in the reference frame of both bodies. Fig. 9 shows the number of body segments and topology of the human dynamic model.

**Figure 10:**
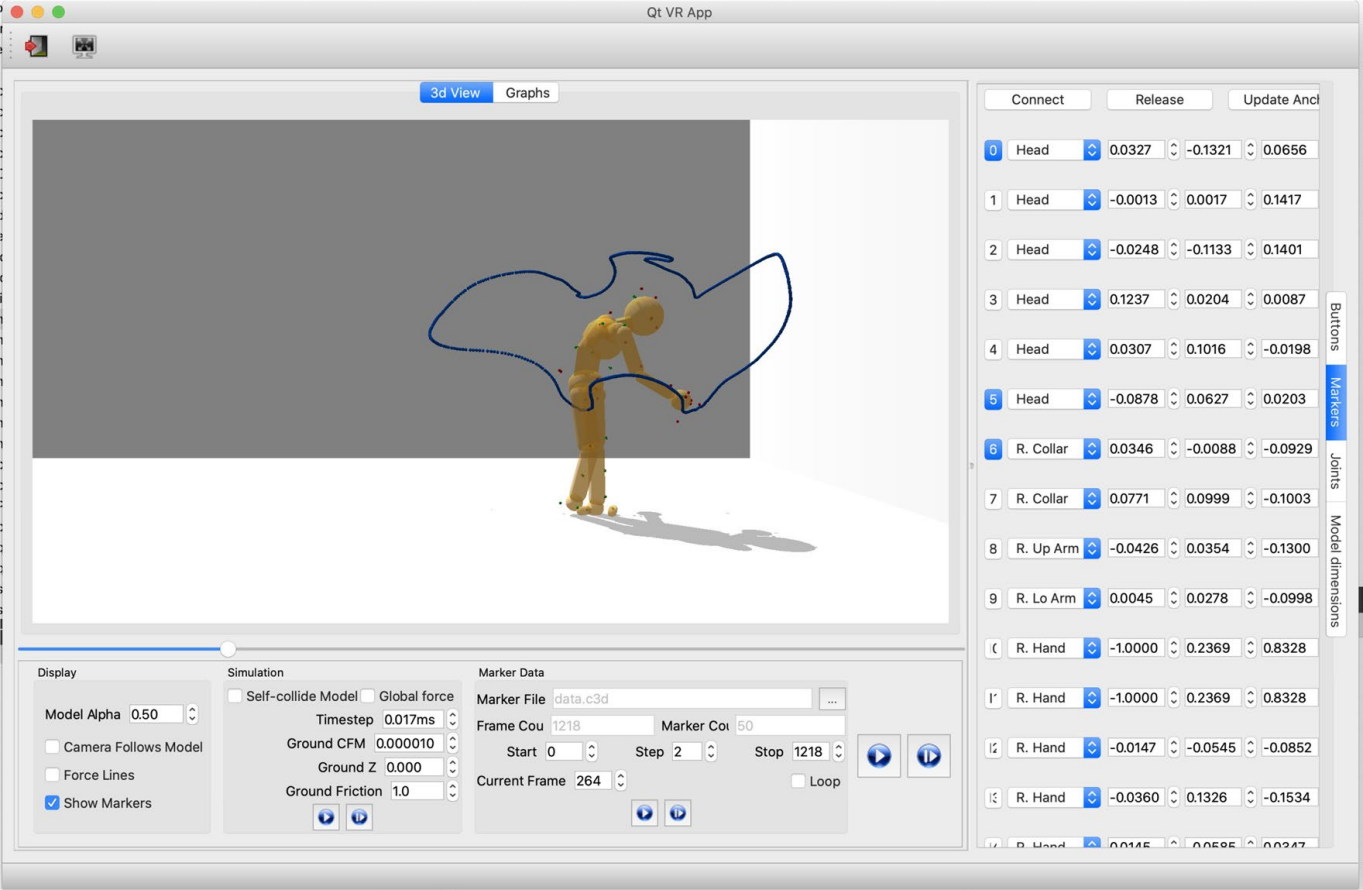
The analysis tool of using the physics engine to compute inverse kinematics and inverse dynamics^28^. The user interface also supports various visualizations of relevant data and control for analyzing and producing physically-based movements. The programmed parameters of the model consist of its joints and its 3D marker positions. For example, the right column represents the positions of the markers relative to their corresponding body segments, e.g. the first row shows the information of marker1: (1) “1” represents the marker index, (2) “head” means marker 1 is attaching to the “head” body segment, (3) the remaining three float numbers are marker1’s relative position.

**Figure 11:**
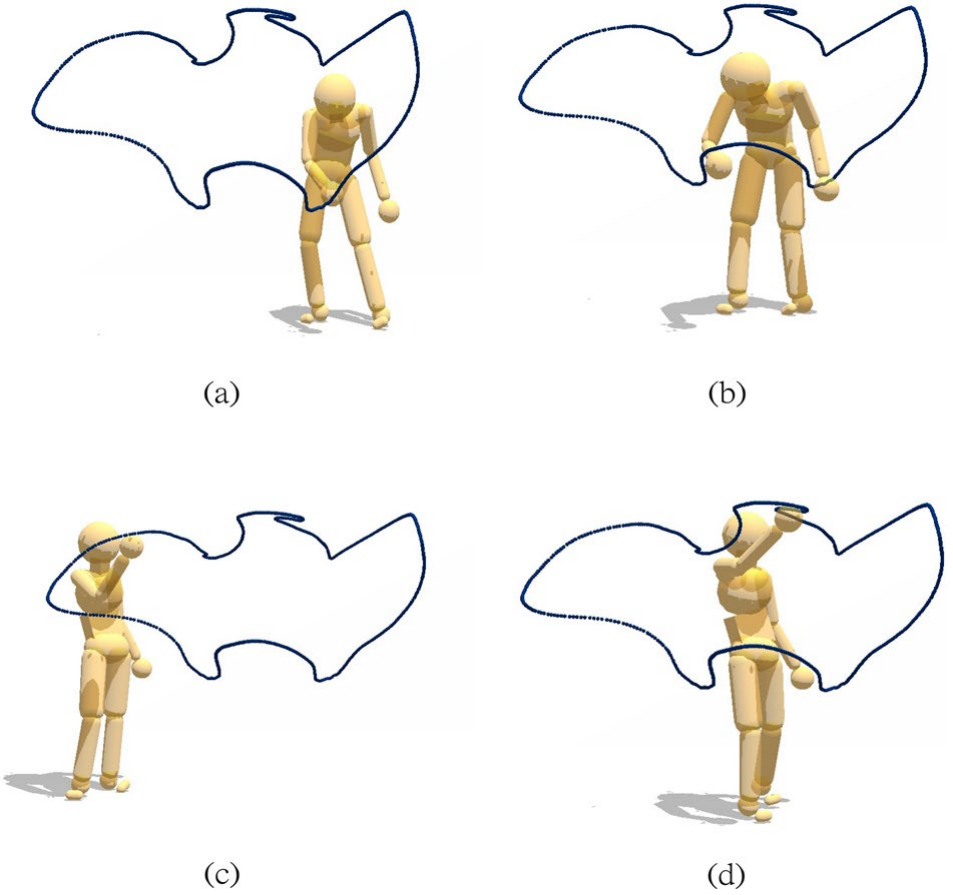
Model capability illustration. Four points in a tracing sequence reproduced with physics-engine-based inverse dynamics using recorded motion capture data from a human subject.

Figure 10 shows a user interface that allows the simulation of human movements via a multi-purpose graphical interface for analyzing movement data captured through interaction with the virtual environment. With this tool, it is possible to interactively fit a model to motion capture data, dynamically adjust parameters to test different effects, and visualize the results of kinematic and dynamic analysis, such as the example in Fig 11, which shows four stages in a tracing sequence made originally by a participant of the virtual tracing experiment and recreated by applying the inverse dynamics method using this tool.

#### Residual forces/torques

The energetic costs are derived from the inverse dynamics technique described in^28^, which combines measured kinematics and external forces to calculate net joint torques in a rigid body linked segment model. A feature of the dynamic method is that it can reduce potential errors, both in the matches of the motion capture suit and the model. Analogous to the human body’s ligament structure to join joints, some leeway is allowed in the model joints in the integration process.

A low cost in residual forces usually implies that the dynamic model is a good match for that subject’s kinematic data. Nonetheless, even after these adjustments, some errors remain. In the model, the main source of the residual forces is usually attributable inaccuracies in the matches between the motion capture suit makers and their match with their corresponding points on the model. We resolve this discrepancy by introducing a coordinate system positioned and the center of mass that cancels the error to maintain balance^28^ and compensates for this problem^70^. As mentioned this resolution with a dichotomy of forces is analogous to the human system, which combines feedforward lateral pathway forces with medial pathway feedback forces.

**Figure 12:**
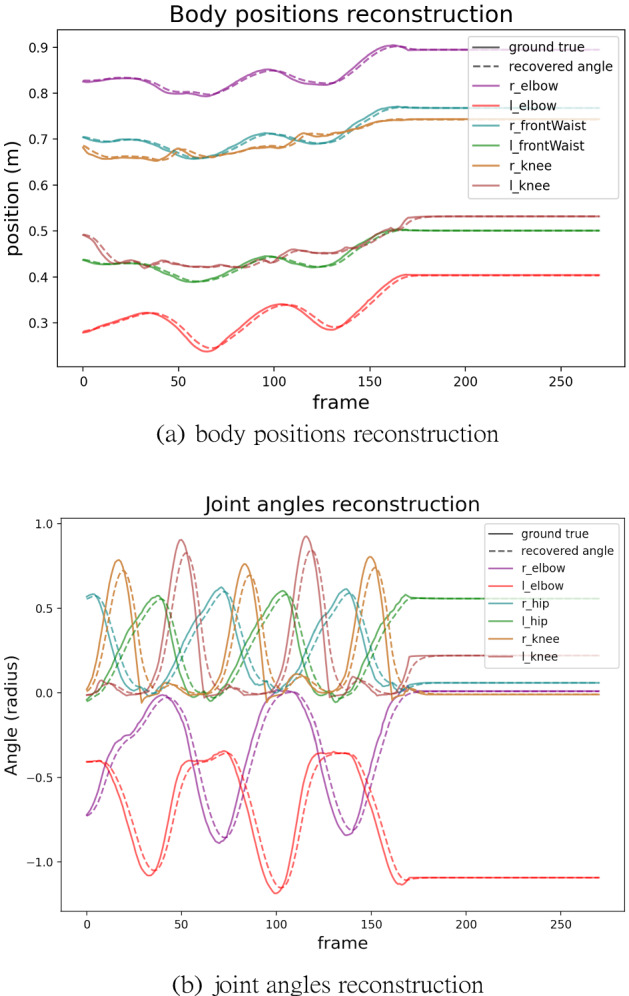
Trajectories reconstruction. The figure shows comparisons between the raw motion capture data shown in the solid lines and the recovered motions in dashed lines along selected dimensions. (**a**) Shows the comparison of body positions along the y-axis. (**b**) Shows the comparison of joint angles along the x-axis.

For example, the comparisons shown in Fig. 12 imply the model does a good job of reproducing the kinematics. Moreover, the solid lines sometimes exhibit some coarse or sharp changes, such as the “l(left)_knee” line around frame 170, caused by some unexpected errors in the motion capture recording system. Surprisingly, the dash lines are pretty smooth even if the solid lines are not perfect. This fact is one of the advantages of the assumption in HDM that the joint constraints behave like springs. However, the disadvantage is that it causes apparent lag and damping of reconstructed trajectories. As a result, the dash lines lag behind the solid lines by a small amount. But the disadvantage will not influence the competition of energy cost.

### Energy cost computation

The centerpiece of the analysis depends critically on the definition of a posture. At each frame, posture is defined as a vector of the joint torques and angles of each of *N* joints ($N= 22$ in our dynamic human model). The posture *p* at a frame is a 6n-dimensional column vector presenting the joints properties of the *i* *th* participant, thus

1 $$\begin{aligned} \mathbf{p}= & {} [\mathbf{j}_\mathbf{1}, \mathbf{j}_\mathbf{2}, ..., \mathbf{j}_\mathbf{N}] \end{aligned}$$

2 $$\begin{aligned} \mathbf{j}_\mathbf{i}= & {} (\varvec{\tau }_\mathbf{i}, \mathbf{a}_\mathbf{i}) \end{aligned}$$

where $\varvec{\tau _i}=(\tau _{i_x}, \tau _{i_y}, \tau _{i_z})$ and $\varvec{a_i} = (a_{i_x}, a_{i_y}, a_{i_z})$ represents the torques and angles of the *i* *th* joint at a frame respectively and $i = 1, 2, ..., N$. For the joints which have less than three dimensions, e.g. hinge joints, universal Joints, the values at unused dimension were assigned zero.

The power *W* of $i_th$ joint at a frame *t* is a scale and equals to the inner product of its torque $\varvec{\tau _i}$ and its angular velocity $\varvec{\omega _i}$, thus

3 $$\begin{aligned} \varvec{ \omega _i}(t)= & {} \varvec{a_i}(t) - \varvec{a_i}(t-1) \end{aligned}$$

4 $$\begin{aligned} P_i(t)= & {} \varvec{\tau _i(t)} \cdot \varvec{\omega _i(t)} \end{aligned}$$

Therefore the power of a posture at frame t is presented as:

$$\begin{aligned} W(t) = \sum \limits _{i=1}^N W_i(t) \end{aligned}$$

Assuming it takes a participant T frames to trace a path, then the total energy cost *E* of the participant tracing a path is:

$$\begin{aligned} W = \sum \limits _{t=1}^T P(t) \end{aligned}$$

The energy cost analysis is naturally organized into three separate stages. Initially, we analyze the subjects energy cost and residual torques of tracing path1 which is the simplest path. Next, we computed the tracing cost of all nine paths. To compare the energy cost of tracing a path across subjects, we computed the average energy cost for all five repeated traces of each subject. Finally, we measured the tracing cost of perturbed participant’s trajectories and perturbed paths.
